# A Probabilistic Model for Estimating the Depth and Threshold Temperature of C-fiber Nociceptors

**DOI:** 10.1038/srep17670

**Published:** 2015-12-07

**Authors:** Tara Dezhdar, Rabih A. Moshourab, Ingo Fründ, Gary R. Lewin, Michael Schmuker

**Affiliations:** 1 Bernstein Center for Computational Neuroscience (BCCN) Berlin, Berlin, Germany; 2 Department of Biology Chemistry Pharmacy, Institute of Biology, Neuroinformatics & Theoretical Neuroscience, Freie Universität Berlin, Berlin, Germany; 3 Department of Neuroscience, Max-Delbrück-Center for Molecular Medicine, Berlin, Germany; 4 Center for Vision Research, York University, Toronto, ON, Canada

## Abstract

The subjective experience of thermal pain follows the detection and encoding of noxious stimuli by primary afferent neurons called nociceptors. However, nociceptor morphology has been hard to access and the mechanisms of signal transduction remain unresolved. In order to understand how heat transducers in nociceptors are activated *in vivo*, it is important to estimate the temperatures that directly activate the skin-embedded nociceptor membrane. Hence, the nociceptor’s temperature threshold must be estimated, which in turn will depend on the depth at which transduction happens in the skin. Since the temperature at the receptor cannot be accessed experimentally, such an estimation can currently only be achieved through modeling. However, the current state-of-the-art model to estimate temperature at the receptor suffers from the fact that it cannot account for the natural stochastic variability of neuronal responses. We improve this model using a probabilistic approach which accounts for uncertainties and potential noise in system. Using a data set of 24 C-fibers recorded *in vitro*, we show that, even without detailed knowledge of the bio-thermal properties of the system, the probabilistic model that we propose here is capable of providing estimates of threshold and depth in cases where the classical method fails.

For many years, pain has been subject to extensive neurobiological, clinical, and psychophysical studies^1^,^2^,^3^. Since the early 20^th^ century when Charles Sherrington conducted neurophysiological experiments that started to define the neural process of detecting and responding to noxious, harmful stimuli as nociception and the responsible afferent neurons as nociceptors^4^, different types of nociceptors have been identified^5^,^6^. The main group includes polymodal unmyelinated nociceptors^7^, responding to potentially painful mechanical and heat stimulation, thus defined as mechano-heat sensitive C fibers (C-MH fibers)^8^,^9^,^10^,^11^. Although C-fibers play a pivotal role in perception of noxious heat stimuli, less is known about the underlying mechanisms that transform heat and mechanical stimuli into neural activity^12^,^13^,^14^.

The pain pathway can be broken down in three major components: 1) transduction of heat or mechanical energy into electrical signals at the receptor site, 2) transmission and modulation of action potentials from peripheral receptor site to the Central Nervous System CNS and higher brain function, and 3) perception of signals as pain^15^. In recent years mathematical and computational models of the pain pathway have been developed and provided valuable insights into various aspects of pain, like acute pain^16^ and neuropathic pain within a dynamical system of neurons^17^,^18^, relating the input stimulation to the sensation of pain in an artificial neural network^19^,^20^, or the temporal dynamics of pain perception using neuroimaging^21^. Recently, mathematical modeling of pain at the cellular level drew attention to the plausibility of physiological properties of nociceptors and bio-thermal properties of skin^22^,^23^. In heat sensitive sensory neurons, the threshold temperature was considered as a determinant for activation of heat sensitive ion channels, and to discriminate nociceptors from warm sensitive neurons. Despite the fact that there at least three ion channels activated by noxious heat are expressed in mouse nociceptors (TRPV1, TRPM3 and anoctamin-1)^24^,^25^,^26^,^27^ there is still no clear picture of where in the skin (e.g. dermal or epidermal free nerve endings) these physiologically important heat transducing ion channels are located. Thus computational models, based on real experimental data, can potentially provide constraints on the thermal changes that a noxious heat transducer must be able to detect in order to transform thermal energy into a noxious heat code in nociceptors. Measuring the threshold temperature of a nociceptor is complicated by the fact that the temperature at the location of receptor differs from the surface temperature, because the skin is not a perfect heat conductor. In addition, the receptor endings are hard to access, due to the complex and variable histological structure of the skin (i.e. several layers, ridges, hair follicles), branching of the free nerve endings of C-fibers, and small diameters of terminal endings. Even the smallest thermocouples are large with respect to the epidermal and dermal thickness and as an external object they would change the conditions of heat flows. An infrared camera could detect the flow of heat, but it cannot reveal the position of nerve endings. Therefore, it is currently not possible to measure the temperature at the transduction site(s) in nociceptor endings directly.

To mitigate these shortcomings, mathematical models were used to estimate the threshold temperature as a parameter^21^,^28^,^29^,^30^. *Tillman et al.*^30^ showed that latency of the first action potential fired by a C-MH afferent depends primarily on the temperature at the location of the receptor (its *depth*), and is hence influenced by the heat conductance of the skin. Therefore, to obtain an accurate estimate of threshold temperature of the receptor neurons, the first step has been to model the propagation of the temperature in several layers of the skin from the surface to the receptor depth. Several models have been developed to estimate nociceptor depth and threshold by using neurophysiological recordings of nociceptors or behavioral measurements to parameterize the heat diffusion equation to model the ability of the skin to conduct heat energy^23^,^30^,^31^,^32^,^33^. In a typical experimental protocol for this purpose, a heat electrode is placed on the skin surface, through which ramped heat stimuli with different rates are applied. The time 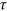 until the threshold is reached can be the time of reporting the sensation of pain in a behavioral experiment^31^,^33^ or the latency of the first action potential in an electrophysiological recording^30^. Assuming that neurons fire at a set temperature, it is possible to estimate the firing threshold temperature and the depth of the receptor from the threshold time 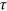 by solving the heat diffusivity equation for different ramp stimuli. The estimated threshold temperature reported in previous studies varies in a range from 39–41 °C^23^,^30^, 43 °C^34^, 45 °C^28^, and 47 °C^35^. However, only in the study by *Tillman et al.*^30^ were threshold temperatures estimated at each neurons’ estimated depth, while the other studies obtained threshold temperature at an assumed fixed depth or at the surface of the skin, which may explain the tremendous variability of the estimates in spite of using the same stimulus parameters.

An accurate estimation of the time at which the threshold temperature was reached is essential for the classical approaches. Small inaccuracies in spike time measurement, internal random process which could result in a delay between reaching the threshold and generation of first action potential, and trial-to-trial variability of neural responses, can result to big inaccuracies in depths, and sometimes even make depth estimation impossible. Additionally, there are many sources of uncertainty in the estimation method. For example, the bio-thermal skin parameters (e.g. thickness, thermal diffusivity) are difficult to estimate accurately, since they vary with the age, gender, recording area and even the hair cycle of the specimen^36^. Moreover, repeated recordings from one neuron are often difficult because of the dynamic experimental conditions when recording from C-fibers^37^. Therefore, the results of the classical approaches to estimate heat threshold and receptor depth have been disputed^22^ and often failed to yield reliable estimates^30^.

In this contribution, we reformulated the established model for temperature propagation in a probabilistic way. We thus open up the way to account for natural variability on receptor response and uncertainties in other model parameters, enabling a statistical approach to the problem of inferring receptor depth from spike times and associated surface temperature. We introduce two modifications to the previous approach. First, we allow for a small, variable delay that can account for the variability of spike latency arising from deterministic properties of the system and/or ‘noise’, e.g. generated by random processes inside the neuron. Second, we use Monte-Carlo methods to deal with the uncertainty in bio-thermal parameters of skin model. To demonstrate the reliability of the new probabilistic model we applied both the classical approach and the probabilistic approach on a data set of extracellular recordings from 24 C-MH nociceptors. We then estimated the receptor parameters, depth and threshold temperature of all neurons and evaluated the probabilistic model using model evidence tests. The proposed method succeeds in producing realistic estimates of both threshold and depth for all measured neurons, although previous methods failed to provide such estimates for all neurons.

## Results

Towards our aim to improve the classical model for depth and threshold estimation we first investigated the factors that rendered previous approaches unstable. We assumed that the instability of the classical model was mainly due to uncertainties of the threshold time and an incomplete skin model. Here, we use the term ‘threshold time’ to refer to the (not directly measureable) time at which the neuron reaches its firing threshold and action potential initiation. In the classical approach, threshold time is assumed to be equal to the time of the first spike. However, the first spike after stimulation onset may be generated with a small delay (latency noise), or it may be caused by spontaneous sub-threshold activity, especially in cases where the spike latency is very short compared to stimulus onset. To deal with these uncertainties and variability in spike times we (i) introduce a delay between threshold time and first spike (latency), (ii) allowing for some responses to be outliers that are not driven by the presented stimulus, and (iii) introduce a probabilistic model for the skin.

### Classical method, directly using the heat transfer model to identify neuron properties

**Figure 1:**
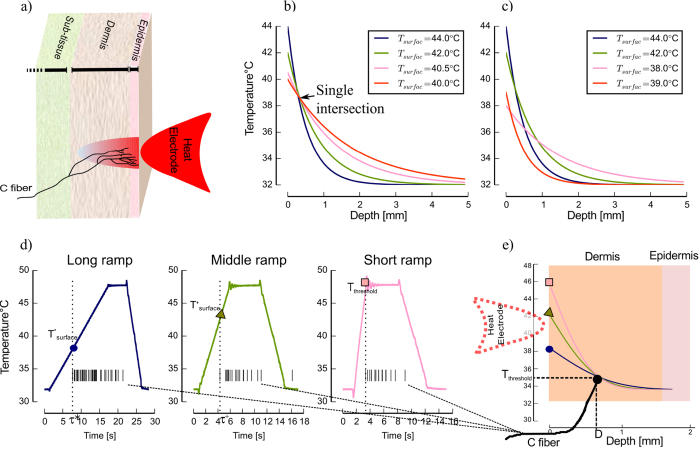
Experimental conditions and schematic of skin model. (**a**) Schematic of skin model and a C-fiber. In a typical experimental protocol a heat electrode is placed on the surface of the skin. (**b-c**) Drift of different surface temperatures for all locations in the skin up to a depth of *D* = 5 mm as a schematic illustration. Each curve refers to a different surface temperature at different time points after stimulation onset of different experimental conditions. (**b**) All four curves intersect in a single point, which refers to threshold and depth of nerve endings. (**c**) There is no single solution of heat equation that is consistent for all four experimental conditions. (**d**) An example of in this study applied experimental protocol. Three heat ramped stimuli with different ramp durations were applied on the dermal side of the skin while the responses of one C-MH nociceptor to all three stimulus conditions were simultaneously recorded. The surface threshold temperatures *T_threshold_* are associated with the first spike times τ for all three conditions. (**e**) Transfer of initial *T_threshold_* of all conditions through the skin layers for the example neuron. The curves intersect at the location of receptor and the same threshold temperature. Note that in our experiments the heat electrode was placed on the inside of the skin.

We first assessed the performance of the classical model to estimate receptor depth and threshold, based on the analytical solution of the heat diffusion equation. An attractive feature of the analytical approximation is that the solution at any point in the skin can be obtained independently from the skin model, which is particularly useful when the skin model is not completely determined, or when the skin is stimulated from the corium side to facilitate electrical stimulation and pharmacological manipulation of the primary afferents in *in vitro* skin preparations^13^. Figure 1a depicts a typical skin model in which the heat electrode is applied on the surface of the skin. The heat energy flows from epidermal to dermal and subcutaneous layers and reaches the free nerve endings of C-MH fibers at some point. The ability of each layer to absorb heat energy and attenuate the surface temperature during transfer depends on its thermal diffusivity.

Assuming a fixed temperature threshold of a neuron at a fixed depth, it is possible to infer this depth from the time until this threshold is reached after applying a constant supra-threshold temperature to the surface. Figure 1b depicts the state of the heat gradients within the skin for four different surface temperatures when reaching a threshold of *T* = 38.8 °C at a depth of *D* = 0.3 mm. Each curve hence refers to a different time point after stimulus onset, i.e. the latency of the first action potential. All curves intersect in a single point: the estimated depth of the receptor.

This idealized example illustrates how the classical approach to depth and threshold estimation depends on reliable spike latency. Neuronal “noise” that causes variability in spike timing unfavorably affects the performance of this method. Real-world measurements affected by noise thus often fail to intersect in a single point, as illustrated in Fig. 1c, thereby failing to provide estimates of location and threshold temperature.

We studied the capability of the classical approach to predict receptor threshold and depth in recordings from 24 C-MH neurons. Our experimental protocol consisted of three heat stimuli with three ramp rate durations of long: 16 s, middle: 4 s, and short (see Fig. 1d for an example). Each stimulus was applied only once and in the same order. The heat electrode was placed on the corium side of the skin, not on the epidermis (see Fig. 1e).

For a given threshold time 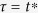 the relation between the surface temperature 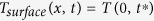 at time *t* *=* t^*^ and the attenuated threshold temperature 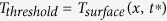 at the location *x* of the receptor can be entered in the solution of heat equation as formulated by Henriques^38^,


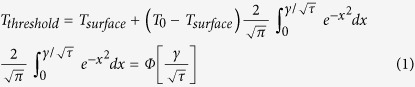


We assume the skin has uniform temperature 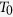 at stimulation onset. In equation (1), we used the short hand notation 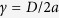, with 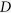 being the receptor depth and 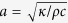 the thermal diffusivity of skin. In the second equality, ϕ denotes the cumulative distribution function of the standard normal distribution. Then all combinations of receptor depth 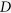
and temperature threshold 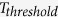 that are consistent with these experimental conditions lie on a curve in the 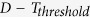 plane (see Fig. 1e). For the same cell under different experimental conditions 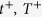 another curve results (Fig. 1d,e). Assuming that the receptor threshold temperature is defined and the same for all three stimulus conditions, then the intersection between these curves corresponds to *D* and 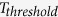 constellations that are consistent with both measurements.

**Figure 2:**
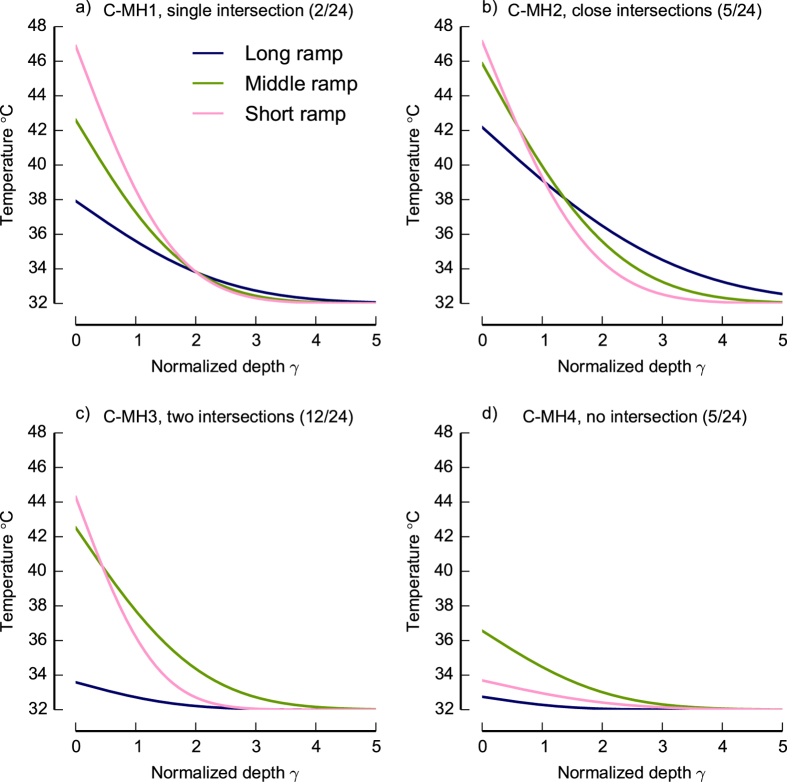
Transfer curves of surface threshold temperature through the skin in a depth vs receptor threshold plane for three different ramp rates and four neurons. (**a**) All three curves intersect in a single point, which is the solution of equation (1) and refers to threshold and depth of this neuron. (**b**) Three curves intersect in three close points and span a triangle. The resulting threshold and depth was determined as an average over three curves. (**c**) Two curves intersect and the third one start at a very low *T_surface_* and remains below the other two. (**d**) The curves start at a very low initial temperature and do not intersect. There is not any single solution for equation (1).

To solve the equation (1) for three ramped heat stimuli we assumed that the threshold time was equal to the latency of the first spike (i.e. the time from ramp onset to the first spike, corrected by electrical conduction delay) for all three ramped stimuli, and measured the temperature at the surface at that time. Figure 2 shows the solution of the heat function and transfer of three surface temperatures 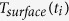, (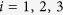 number of stimuli) in 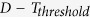 plane for 4 example C-MH neurons.

Only 2 out of 24 neurons intersected in a single point (Fig. 2a shows an example), 5 out of 24 intersected in three close points and spanned a small triangle (e.g. Fig. 2b). For 12 out of 24 the temperature curves which correspond to two ramped stimuli intersected in one point but the third curve did not intersect the other two curves (e.g. Fig. 2c). For the remaining 5 neurons the curves did not intersect at all (e.g. Fig. 2d).

For the 2 neurons with a single intersection point distinct depth and threshold temperature were directly obtained by the intersection. For 5 neurons that the intersections span a small triangle we generated an average over the intersections. For cases where the intersections span wider ranges, generation of an average over stimulations induces a high variance in the estimated depth, leading to an expected large estimation error for threshold and depth.

The mean average receptor threshold temperature for the 2 neurons with a single intersection was 39.44 °C and standard deviation of 5.7 °C, consistent with values found in the literature^30^. Yet, for the majority of neurons (17 out of 24) the classical method could not determine a threshold estimate of all three ramped stimuli, pointing out the potential for improvement of this classical approach toward a better estimation of depth and threshold.

The main limitation of the classical approach lies in its assumption that parameter constellations are either fully consistent with the experimental outcome (i.e. they are on the curve) and can be included in the estimation or they are completely inconsistent with the experimental outcome (not on the curve) and might be discarded. In the following sections we will use equation (1) as part of a probabilistic model, which allows us to formulate a continuous measure of consistency with the experimental outcome (a likelihood function). This allows us to identify a unique most likely combination of receptor depth and temperature threshold.

### Probabilistic method, Likelihood function

The classical method for inferring depth and temperature threshold assumes that the latency of the response is exactly equal to the time when the temperature threshold was reached. Here, we weaken this assumption by introducing a delay, 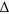 between the threshold time (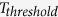) and the response latency (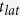). Thus we now evaluate equation (1) at 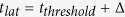. For measured responses to three ramped stimuli, we then have,


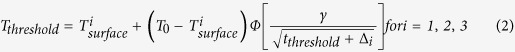


In this equation, 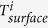 denotes the surface temperature at time 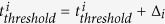 in the *i*-th measurement and 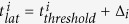 is the latency of the response in the i-th measurement. Equation (2) is a system of *n* = 3 equations with the same parameter 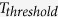 and *γ* but *n* = 3 different 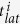 and 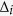 of three stimuli conditions. As we have shown in previous section it is not generally possible to determine a single intersection point. However, because 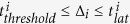, we can postulate that the delays should be (i) positive and (ii) small. We will therefore assume that 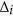 are exponentially distributed with a rate parameter λ. In our experience the exact value of λ did not change the results, so that we fixed λ = 1 for all of the following. With the given data, we can write Δ_i_ as a function of 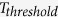 and *γ*, by inverting equation (2).

This allows us to write the log likelihood function;


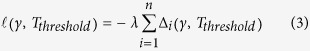


The parameters 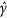
and 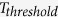 that jointly maximize this function are maximum likelihood estimates of the underlying neuron parameters. After finding the estimate for 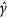, we can derive the depth using the equation 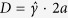.

We can view the negative of the right side of equation (3) as an error function that we attempt to minimize under the constraint that 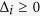. The smallest error can be attained if all delays are exactly zero in which case this method agrees with the classical method discussed in the previous section. In cases in which no unique parameters setting exists, the assumption that responses should be instantaneous, is relaxed by letting one or more 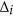 be larger than zero. Note however that increasing the delays does also increase the error. Thus, the parameter constellation with minimal error will be one that is consistent with the data by assuming only minimal response delays.

**Figure 3:**
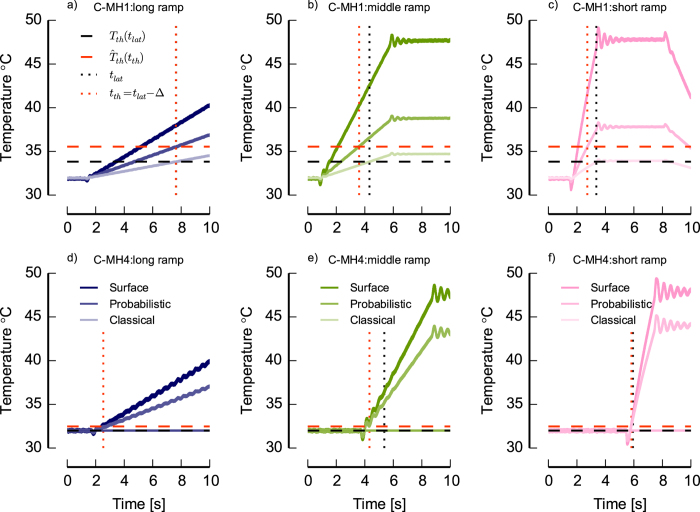
The measured thermal stimulus traces applied on the surface of the skin with three ramp rates and the estimated stimulus traces within the skin at the estimated depth of neurons’ terminals for two example neurons C-MH1 and C-MH4. The estimation of the depth and *T_threshold_* was done according to the classical and probabilistic method. The probabilistic estimated *T_threshold_* is the solution of equation (3). The classical *T_threshold_* is the intersection of *T^i^_surface_*, *i* = 1,2,3 curves in *D-T*_threshold_ plane for three ramp rates of equation (1). (**a–c**) The stimulus traces of neuron C-MH1 for (**a**) long ramp, (**b**) middle ramp, and (**c**) short ramp. Both classical and probabilistic methods successfully estimated the parameters. The classical model approached a single intersection of ramped stimuli and probabilistic method approached small delays. (**d–f**) The stimulus traces of neuron C-MH4 for (**d**) long ramp, (**e**) middle ramp, and (**f**) short ramp. The classical method failed to find an intersection but the probabilistic method resulted successfully to a parameters estimate.

Figure 3 shows, for two example neurons C-MH1 (Fig. 3a–c, single intersection, in Fig. 2a) and C-MH4 (Fig. 3d–f, no intersection in Fig. 2d) the stimulus traces for three different ramp rates (long-, middle- and short ramps) at the surface together with the attenuated stimulus traces at the classically estimated receptor location 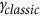 and at the probabilistic estimated receptor location 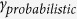.

In example neuron C-MH1 the classically estimated normalized depth is 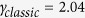 and probabilistic estimated is 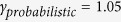. As Fig. 2a shows for C-MH1 the classical model results successfully in a single intersection. The probabilistic estimated location of neuron, however, is closer to the surface compared with the classical estimate. As a result the estimated stimulus at 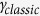 is weaker for all three ramp rates.

Note that in Fig. 3a the estimated delay for the longest ramp is close to zero but not for the middle ramp (Fig. 3b) and short ramp (Fig. 3c).

In the case of neuron C-MH4 the classical method failed to find an intersection of all three ramp stimuli (see Fig. 2d) and therefore the stimulus traces could not be tracked at location 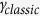. But the probabilistic method estimated successfully a depth and thus the stimulus traces could be estimated at this location (see Fig. 3d-f). Note that neuron C-MH4 shows a small latency for all three ramp stimuli. The first spike in all three conditions occurred immediately after the stimulus onset. This observation may suggest that the first spike may actually have been caused by sources of activity other than the onset of the heat ramp.

**Figure 4:**
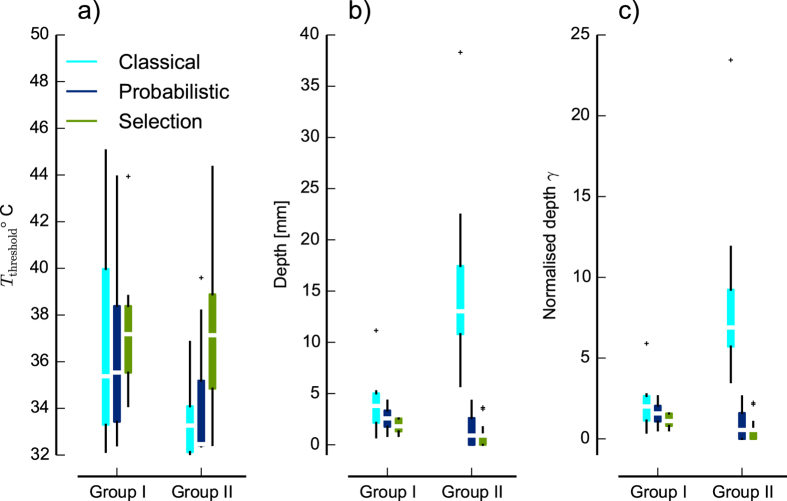
Estimated threshold temperature *T*_threshold_, depth D, and normalized depth *γ* of 24 C-MH neurons according to three methods: classical model, full probabilistic model and a selection model. For the selection model, in which the outlier curve was omitted, we chose the model with the highest evidence. Neurons are divided into two groups. In group ‘Single intersection’ or ‘Group I’ the classical model was successfully resulted in a single or very close intersection. This group includes 7 neurons. In ‘Two/None intersection’ or ‘Group II’ the classical model failed to estimate an intersection. This group includes 17 neurons. The boxes extend from the lower to upper quartile values of the estimated in each group, with a white bar at the median. The whiskers show the range of the estimates. Flier points are those past the end of the whiskers.

Figure 4 summarizes the results of analysis of all neurons for three estimates 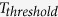, depth 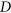 and normalized depth *γ*. We divided all 24 C-MH neurons into two main groups: The group ‘Single intersection’ that includes 7 neurons for which the classical model successfully resulted in a single intersection or the intersections span a small triangle like neurons C-MH1 and C-MH2 of Fig. 2a-b. We refer to this group as ‘Group I’. The group ‘Two/None intersections’ that includes the remaining 17 neurons for which the classical model failed fully or partly to estimate any intersection like neurons C-MH3 and C-MH4 of Fig. 2c-d. We refer to this group as ‘Group II’. The estimated parameters in ‘Group I’ for both classical and probabilistic models compared with the same parameters in ‘Group II’ reveals that in ‘Group I’ both approaches were successful. But, the classical model shows very implausible values for the ‘Group II’: a median threshold temperature of *T*_*threshold*_ = 33.3 °C and mean depth of *D* = 12.5 mm. Previous studies have shown that the majority of C-MH fibers display a threshold of 43 °C^2^,^34^, but several studies have reported threshold temperature in a range of 36-47 °C^28^,^30^,^39^,^40^. Given the thickness of the skin <1 mm in our experiments and the reported values of threshold temperature, the classical model failed to estimate the parameters for the ‘Group II’ with two/none intersections. The full probabilistic model shows for ‘Group II’ a mean threshold of *T*_*threshold*_ = 32.36 °C and a mean depth *D* = 0.9 mm. Hence this model likely failed to estimate *T*_*threshold*_ (because it is implausibly low), but the estimated depth was consistent with the reported depth from the previous studies in range of 20-570 μm^30^,^39^. Note that in our experimental setup the heat electrode was placed on the inside of the skin, so the depth of the receptor from the epidermis was *D*_*e*_ = 100 μm (assuming that the skin is approximately 1 mm thick).

As a brief summary, in this section we showed that using a statistical model to estimate the parameters gives a successful parameter combination of depth for all neurons even when the classical model fails to give any parameter estimate. Yet, in the case of estimated threshold temperatures the values with a median of *T*_*threshold*_ = 32.36 °C were lower than previously reported thresholds in range of 36–47 °C^28^,^30^,^39^,^40^. We then used the calculated depth to estimate the attenuated stimulus traces at the location of the receptor ending.

The major contribution of the probabilistic model is to incorporate noise by allowing for delay in first spike time. However, we didn’t cover the possibility that the first spike recorded after stimulus onset might be an ‘outlier’ which was not triggered by the stimulus, but rather generated by the (low) resting state activity of the receptor. Next we therefore addressed the question of how our results would change under the assumption that the first spike after stimulus onset may have been triggered by a probabilistic random process.

### Selection method, discarding outlier responses

Every stimulus that is taken into account for the estimation of depth and threshold adds a certain amount of information and reduces the uncertainty of the estimation. But since there is also a certain probability that the first spike is not a response to the stimulus, every stimulus also adds a certain amount of noise to the estimation. Hence, there exists a potential tradeoff in adding as many recordings as possible to the estimation, or rejecting some of them as outliers. For example in our data for 12 out of 24 neurons in 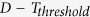 plane, two out of three curves intersect in one point, while the remaining curve either intersects in other points or does not intersect. Essentially, at least two ramp stimuli of different slope are required to observe an intersection of the curves. But, we asked if we reduce the variability of the estimate in the case of widely spanned ranges of intersections by omitting some curves.

We extended this idea to the maximum likelihood framework described in the previous section. We introduced multiple probabilistic models that could describe the data and then decided which one provides a more concise description.

To discriminate between the models we employed a Bayesian testing procedure^41^ and used model comparison based on marginal likelihood^42^. This approach is usually applied to pairwise comparisons: to compare two models, the marginal likelihood of each model is evaluated. The ratio of the marginal likelihoods from model 1 and model 2 is called the Bayes Factor. If the ratio of marginal likelihoods from model 1 and model 2 is larger than 1, the first model is preferred and vice versa, the second model is preferred.

The main reason for adopting a Bayesian approach for model comparison is that Bayes factors provide a way of including other information as prior knowledge when assessing the evidence for a hypothesis. This is a strong advantage for data with lots of uncertainties, such as a small number of recorded neurons and no repetition of recordings for each stimulus, or an incomplete skin model. We can incorporate histological and bio-thermal information and results reported by other investigators as prior knowledge and use this to improve the estimation of receptor parameters. Furthermore, by integrating over the full parameter space, marginal likelihoods penalize complex models in a very natural way^42^,^43^.

Bayes Factors can only be calculated for pairwise comparisons. Yet, here we are interested in selecting the best out of multiple models. We therefore used the marginal likelihoods directly and chose the model that maximizes the marginal likelihood. For simplicity, we will refer to any of the models that exclude one or more stimuli from the determination of the neuron’s parameters as the ‘Selection model’.

The first model is the full probabilistic model described above, assuming a data set consisting of *n* different experimental conditions, e.g. ramped stimuli, where for all responses the first spikes have been triggered by the stimulus. More specifically, the first model assumes that the corresponding delays 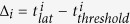 (*i* = 1,2,3 number of stimuli) have a probability distribution with free parameters 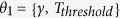. Here, the unknown skin parameters and other nuisance factors could go into 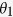 as well.

In the ‘Selection models’ only for the first *m* < *n* responses the first spikes have been triggered by the stimulus and would therefore contribute meaningfully to the estimate of depth and threshold. For the remaining responses the first spikes are the result of a stationary background process, e.g. spontaneous discharges or in any other way not triggered by the stimulus. Thus the second model assumes that only the first delays 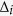, *i* = 1,…,*m*, and *m* < *n* have the same probability distribution with parameters 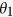 and the remaining delays are based on processes with different distribution functions. This second model has a parameter vector 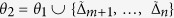 that contains the parameters from the first model and some additional parameters to capture the times of the non-stimulus-triggered first spikes.

In the case of our data the delays of three ramped stimuli in first model M_1_ have the same probability distribution with the parameter space 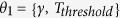. In instances of the selection models M_2_ only two delays out of three have the same distribution with the parameter space 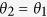
and the third delay has a density function 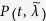 with parameter 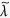.

To compare the models, we use the marginal likelihood,


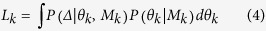


In equation (4), 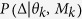 is the likelihood of observing the delays given the parameters 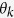
under model 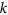. Making the dependence of the likelihood on the model explicit here illustrates that in addition to the parameters of the model, the likelihood of the data depends on the formulated model itself as well. The term 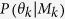 is the prior density of the parameters of model M_k_. This term provides a way to include other information about plausible values of parameters. Our first concern is thus how to choose prior densities to represent the available information.

The prior densities offer an appropriate way of adding biologically meaningful constraints from the literature into a statistical model. In Table 1 we show a summary of reported parameters and the prior density functions we employed here (for more details of choice of prior densities see section methods).

We applied the ‘Selection models’ on the 24 neurons. To demonstrate the performance of the selection models, we chose the model M_2,i_ (*i*-th stimulus was omitted) with the highest evidence and compared the estimates with the results of full probabilistic model M_1_ and with the classical model. Figure 4 shows the estimated 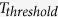, depth and normalized depth *γ*. The selection model shows for the ‘Group II’ a mean threshold of 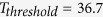 °C and a mean depth *D* = 0.8 mm. In contrast to the full probabilistic model, the selection model approached successfully to a mean threshold temperature, which is consistent with the previous studies.

**Figure 5:**
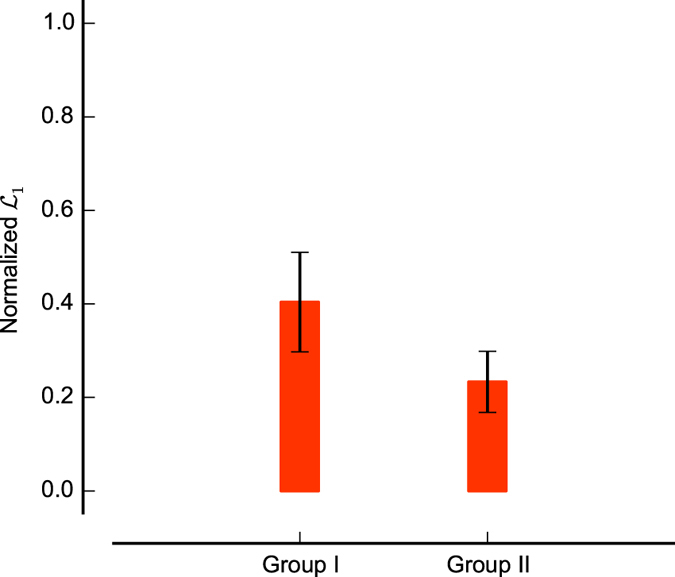
Normalized marginal likelihood for the full probabilistic model M_1_ for two groups of neurons. The ‘Group I’ includes 7 neurons that could be estimated successfully by classical model. The ‘Group II’ includes the remaining 17 neurons. The error bars are the standard error of the mean.

We supposed that the main reason why the classical model failed was its inability to account for neural noise and for statistical outliers in the responses. Thus, one might expect that a working stimulus selection model would mainly detect outliers, and thus exclude the response to one stimulus from the parameter determination for those neurons for which the classical model failed. To test this idea, we normalized the sum of the marginal likelihoods for each neuron across all candidate models to 1. We then plotted the normalized marginal likelihood for the full model that does not exclude any stimuli (see Fig. 5). Clearly, the marginal likelihood *L _full_* was larger for the ‘Group I’ for which the classical model had been successful than it was for the ‘Group II’ for which the classical model had failed to find a consistent parameter estimate. Put another way, this indicates that for many neurons in ‘Group II’, one of the selection models provided a better description than the full model. As the selection models accounted for outliers, while the full model did not, we conclude that statistical outliers might have contributed to the failure of the classical method on the neurons in ‘Group II’.

Thus, the results from our method suggested that for ‘Group II’, selection models that accounted for statistical outliers were more successful at describing the data, suggesting that outliers had indeed contributed to the failures of the classical method in these neurons.

## Discussion

In this study we have shown that considering variability, noise and stochastic processes in study of primary afferent pain receptors improved the classical deterministic models to estimate the depth and threshold of C-fiber nociceptors. We have shown that allowing for a small delay in the first response of C-MH nociceptors modified the localization of the receptor neurons. Moreover, assuming some stochastic activities which were not triggered by stimulus and discarding the outlier responses caused by these stochastic activities further improved the estimation of activation threshold of C-MH neurons. We demonstrated the reliability of our general framework on a challenging data set, in which the stimulus electrode was placed on the corium and dermal side of the skin, the thickness of measured skin was unknown and each stimulus condition was applied only once. Our approach provides a unique insight into how temperature impinges on heat transducers in their native, complex environment. Most studies have hitherto focused on activation of channels by membrane heating in an *in vitro* context where skin is absent^44^. Our study provides a framework to understand where and with what temperatures native heat transducers are activated *in vivo*.

Introducing delay in response time allows us to weaken the assumption that latency and the threshold time should be the same. However, a delay is justified only if the probabilistic model converges to the classical model in boundary condition. Because in classical model all delays are neglected it describes an ideal, noise-free case and thus represents the boundary condition for the probabilistic model when delays are zero. Hence in the boundary condition the classically estimated model parameters should maximize the log likelihood function of delays. Our results confirmed that for negligibly small delays, the probabilistically estimated parameters converge towards the classically estimated results. Hence, the probabilistic model improves the results in cases where the classical model failed and converges to the classical model in boundary condition.

An alternative way to account for noise in the system might be to add white noise into equation (1) and treat it as suggested in the classical method. However, this approach would require to either predetermine the amount of the noise, or to assume that noise is generated by a random process and systematically vary the parameter governing that process. Because of the unknown sources and amount of noise, the first method might add more uncertainties to the system and the latter method might result in a biological meaningless source of noise.

In fact, while the probabilistic model improved the estimation of depth, it did not improve the estimate of the mean threshold temperature. The low average threshold temperature around the baseline temperature for some results estimated by probabilistic model suggests that for some responses, a small delay in threshold time might not be sufficient to deal with the noise. Indeed, the estimation of depth and threshold temperature was improved by discarding these outlier responses in the ‘Selection models’. In addition, the marginal likelihood ratios suggested that the ‘Selection models’ described the observed latency better than the initial probabilistic model that used all stimuli to estimate the neuron’s parameters. Using marginal likelihoods also offered a way of adding prior information about model parameters from the literature by choosing an *a priori* density function. For some parameters, there were multiple possibilities for choosing the prior density. This could have influenced our computation of model evidence^42^. Yet, when we repeated our analysis with slightly different priors, the estimated model evidence consistently favored the same model (for more details see section methods; choice of prior density functions). We therefore believe that our outlier-detection method is relatively insensitive to the choice of the exact parametric form of the prior distribution, as long as the range of values from the literature is captured.

Localization of sensory endings is particularly interesting for quantitative models that describe the responses of a neuron to stimulation and for attempts to uncover the neurons encoding strategy. For instance, in mathematical modeling of touch sensitive A-fibers several investigators have used the histologically measured depth to model the somatosensory responses^45^,^46^,^47^. However, the thickness of epidermis and dermis is variable over a population of animals and depends on the gender, age, area and hair cycle, and anatomical location^36^. In many experimental setups the thickness of skin layers and location of neuron have not been measured. Even in such cases our method can be freely adapted to estimate the depth and threshold of neurons by replacing the heat stimulation with mechanical stimulation and the heat transfer function with the stress/strain functions. Whereas other investigators required complex and detailed skin models, we presented a simple probabilistic model of skin. This way, our method can provide results for cases in which the data do not warrant the formulation of a very detailed skin model, or where insufficient information about the thermodynamic properties of the studied tissue is available.

## Methods

Experiments were performed in strict accordance with the recommendations in the Guide for the Care and Use of Laboratory Animals of the Max Delbrück Centre for Molecular Medicine. The protocol was approved by the Committee on the Ethics of Animal Experiments of Animal Welfare German authorities (LaGeSo; Permit Number: T (00383/12).

### Skin nerve preparation and identification of single C-fibers

The skin-nerve preparation was used to record from single primary afferents^48^,^49^,^50^,^51^. Mice were killed by CO_2_ inhalation for 2–4 min followed by cervical dislocation. The saphenous nerve and the shaved skin of the hind limb of the mouse were dissected free and placed in an organ bath at 32 °C. The chamber was perfused with a synthetic interstitial fluid (SIF buffer) the composition of which was (in mM): NaCl, 123; KCl, 3.5; MgSO4, 0.7; NaH2PO4, 1.7; CaCl2, 2.0; sodium gluconate, 9.5; glucose, 5.5; sucrose, 7.5; and HEPES, 10 at a pH of 7.4. The skin was placed with the corium side up in the organ bath. The saphenous nerve was placed in an adjacent chamber on a mirror to aid and under microscopy fine filaments were teased from the nerve and placed on the recording electrode. Electrical isolation was achieved with mineral oil.

**Figure 6:**
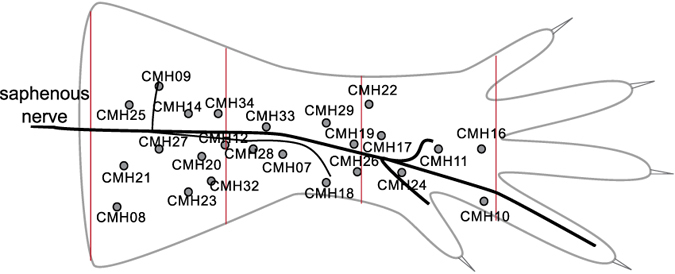
Schematic of hind-limb skin of mouse and anatomical locations of receptive field. For all 24 neurons the locations of the receptive fields are illustrated in respect to the saphenous nerve trunk.

Single mechanically sensitive units were characterized by probing the skin with a glass rod for mechanically responsive receptive fields. Figure 6 shows the anatomical location of all 24 neurons relative to the saphenous nerve trunk. Using an electrical stimulating electrode, we measured the conduction velocity (calculated by dividing conduction distance over electrical latency for the spike) to select C-fibers whose conduction velocity was in the C-fiber range <1 m/s.

### Thermal stimulation protocol

For heat stimulation, a Peltier element–based contact probe (4 × 3 mm surface) applied thermal stimuli (custom device built by the Yale School of Medicine Instrumentation Repair and Design) to the receptive field of identified C fibers.

The stimulation protocol sent as a pre-programmed series of commands to the Peltier element were applied to all C-fibers. The thermal stimulation protocol consisted of 3 stimuli (32–48 °C) with different ramp durations (16 s, 4 s, 2 s) and the hold phase of 10 seconds. The interstimulation interval was 60 seconds. The order of stimulation was first heat and then mechanical from the slowest to fastest ramps. After the thermal stimulation we carried out a mechanical stimulation protocol, the results of which are not part of this manuscript. Data were obtained from 11 C57Bl6/N wildtype mice.

### Heat transfer model of skin

Heat energy flows through the skin to the nociceptive terminals due to the temperature gradients. According to the second law of thermodynamics, heat flows from warmer location to colder location. With no heat source, these thermodynamical effects will let the skin relax to one homogeneous temperature. In an experimental set using a Peltier device, the stimulus acts as a heat source, inducing heat at one location which then flows through the skin and eventually results in a temperature increase at the receptor site of neuron. Because the stimulus temperature varies over time, the resulting heat flow will vary over time as well. In addition, it varies in space because skin is not an ideal conductor and locations further from the stimulus receive lower heat energy and at a later time. Directly measuring the exact temperature profile in the skin at the depth of nociceptor is difficult. Therefore, heat transfer in the skin is typically modeled using thermodynamical laws^32^,^38^. The general equation describes heat flow in the skin by a diffusion equation,


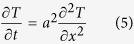


Here, 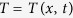 is the temperature at time 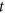 and distance 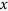 from the stimulus contact, such that 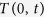 is the experimentally applied heat stimulus and the temperature at other locations needs to be determined by solving equation (5). Furthermore, 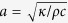 is called the thermal diffusivity. Thermal diffusivity is square root of the ratio between thermal conductivity 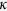 and the product of density 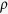 and specific heat 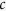 of the skin.

When solving equation (5) analytically, one has to assume that the skin is a solid with uniform thermal properties. Given the complex structure of the skin, this assumption is likely to be violated. More realistic approaches therefore treat the skin as consisting of multiple layers, where each layer has uniform thermal properties but layers are allowed to differ in their thermal diffusivity. Typical models use separate layers for epidermis, dermis and subcutaneous tissue^30^,^32^. Other models have used four layers^38^ or up to eight layers^28^ in live animal models. In all these multi-layer approaches, the stimulus is assumed to be applied at the epidermal side of the skin and the thickness of each layer has to be specified in advance. Then, a system of equations describing the flow of heat into and out of each of the layers can be derived^30^,^33^. used a modified finite difference schemes to numerically solve these equations, while^31^,^38^ derived a first order approximation of the time-temperature relationship in every layer and at any distance from the skin surface according to the equation (1). This approximation was originally derived for experiments in which the stimulus is abruptly switched on and is then kept fixed until the threshold is reached. However, these approximations have also been successfully applied to conditions where the surface temperature is not constant but is known at every instant in time^31^. This perspective can be justified if we assume that heat conductance in the skin is sufficiently fast. Then the temperature at any distance can be computed quite accurately until the steady state temperature is reached.

### Estimation of depth from *γ* using a Monte-Carlo method

Several investigators measured the epidermal and dermal thickness and their thermal properties. In Table 1 we summarized some measurements. So far the experiments don’t differ, we would start usually with epidermis as superficial layer and estimate 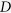 for a determined diffusivity of epidermis according to,


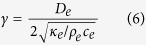


If the estimated 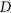 does not exceed the histological measured epidermal thickness, we would accept it as the depth of nerve endings, otherwise *γ* would compose of epidermis and dermis layers


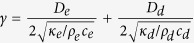


Where, *D*_*e*_ is the depth of the epidermis, and *D*_*d*_ is the depth of dermis. To estimate *D* in equation (6) we would then determine *D*_*e*_and thermal diffusivities and solve the equation for *D*_*d*_.

In our data set, however, the heat electrode was placed on the dermal and subcutaneous site of the skin and the thickness of the skin wasn’t measured. This experimental redundancy prevented using a direct multilayer model of the skin. Therefore we reduced a two layer model with separate layers for epidermis, and dermis to a one layer model, which accords properties of both epidermis and dermis. Next step is to set the skin of a one layer model. To this end, we took the maximum and minimum reported values of thermal conductivity, density, and specific heat of epidermis and dermis from literature (see Table 1). We then sampled three sets for 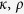 and 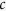 from respective uniform distributions. The sampling range of the distributions were the maximum and minimum values for epidermis and dermis. For the estimated normalized depth, 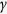, we drew every single parameter from the corresponding sampling distribution and computed the depth. We then repeated this procedures for the whole sampling sets with size of 10^8^ and in the end averaged over the computed *D*.

### Choice of prior density functions

In order to express the prior assumptions about the value of 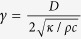, we need an *a priori* density function of the depth and all parameters of the thermal diffusivity. According to histological studies most C-MH fibers end in the epidermal to dermal layers of the skin^52^. Therefore, the probability of finding the nerve endings decreases with increasing depth, but it is unlikely to find nerve endings directly beneath the skin surface. Furthermore, depth cannot be larger than the skin’s thickness, which is in our experiments approximately 500 μm to 1 mm. Note that in our experimental set up the position zero refers to some distances deep into dermis and subcutaneous tissue. The shape of this distribution seems to have a probability density function with asymmetric tails but a heavier tail toward zero. A distribution with this property is offered by Beta distribution. Beta distribution has two shape parameters α and β. These shape parameters are determined if two properties of the distribution are known, for example the mode and a given percentile. In previous studies the locations of C-MH fibers were estimated in the depths ranging from 20 to 570 μm (see Table 1 for references). Therefore, we scaled the Beta distribution in an interval of 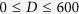 μm and assumed that the depth of a receptor neuron had a Beta distribution with 75-80 percent of the distribution’s mass in an interval around median of an approximate width of 400 μm and mode 
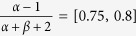
. We arbitrary chose two shape parameters α = 4.75 and β = 2.25 which satisfied these assumptions. In addition and to verify that this choice of prior did not influence our analysis, we also ran the same analysis with other shape parameters and a uniform and Weibull distribution.

We chose uniform distribution U(a,b) (with a and b being the minimum and maximum values of distribution) for the thermal diffusivity parameters. To this end 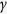 composes of four distribution functions in form of 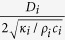 and 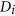 ~ B(4.75,2.25), 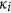 ~ U(0.3,1.3), 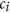 ~ U(700,950), and 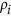 ~ U(1116,1200), (for the *i-*th draw from sampling population).To define an explicit density function 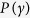 we firstly drew samples 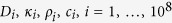 from the respective distributions and calculated 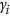. It is very difficult to derive an analytical form for the distribution of *γ* from these assumptions. Therefore, we approximated the distribution of the sampled *γ* by a mixture of 3 Gaussian distributions with unknown parameters (estimated using expectation-maximization (EM) algorithm for fitting mixture-of-Gaussian models implemented in scikit-learn, the number of components was determined by minimizing Bayesian Information Criterion (BIC).).

A more complicated case is the prior 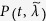 for the outliers 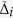 in ‘Selection models’ M_2_. In order to create a meaningful marginal likelihood, the prior should be a proper probability density function^42^. In addition, the priors should be random statistical process that generate the first spikes independently of the stimulus. An example density that satisfies this property assumes that the first spike times are spontaneous events, which are generated by a Poisson process. Under these conditions the first spike time is a random event of an exponential distribution with a rate parameter 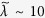. To estimate the total evidence for a model, we took the expectation with respect to 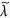. This expectation was implemented as a numerical integration.

In Table 1 we summarized all priori distribution functions for parameters from literature including the references.

### Calculating the marginal likelihood

Typical strategies to estimate the marginal likelihood (4) are sampling (i.e. Monte-Carlo integration) or analytical approximations like the Laplace approximation, Variational Inference, or Expectation Propagation. For low dimensional parameter spaces (2-4 dimensions), numerical integration may work as well. In equation (3), the delays are only given implicitly, which makes analytical approximations quite difficult. Furthermore, numerical integration techniques would be computationally very intensive due to the cost of solving equation (3). We therefore used a combination of analytical and numerical integration to determine the marginal likelihood. We interpreted the integral (4) as the expected value of the likelihood function 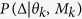, under the prior density 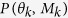 for model M_k_,


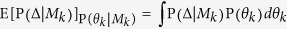


and used numerical integration according to trapezoidal rule to compute the expected value. The prior densities are for model M_1_: 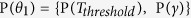 and for model M_2_: 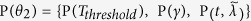. For ‘Selection models’, M_2_, however, we assumed the outlier first spike has a fixed value, so that we can formally write the likelihood for the outlier components as a dirac function 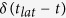, which allowed us to solve for the corresponding components of the marginal likelihood (4) analytically. All other parameters of model M_2_ were treated in the same way as in M_1_.

For every potentially excluded stimulus in selection model M_2_, we estimated the ratio of the marginal likelihood for the full model M_1_ over the marginal likelihood for the model M_2_ without the excluded stimuli.

## Additional Information

**How to cite this article**: Dezhdar, T. *et al.* A Probabilistic Model for Estimating the Depth and Threshold Temperature of C-fiber Nociceptors. *Sci. Rep.* **5**, 17670; doi: 10.1038/srep17670 (2015).

## Figures and Tables

**Table 1:** Priori distributions for different parameters.

Parameters	Reported value	Subject	Reference	Prior density	Remark
Threshold	36 °C	Human	40	N(38,2)	First sensation
	39–41 °C	Monkey	30	N(38,2)	First spike
	43.2 °C	Human	31	N(38,2)	First pain
	43 °C	Human	39	N(38,2)	–
	45 °C	Human	28	N(38,2)	–
	47 °C	Rat	35	N(38,2)	–
Depth ***D***	20–570 μm	Monkey	30	B(4.75,2.25)	C fibers
	180–240 μm	Human	31	B(4.75,2.25)	C fibers
	100 μm	Human	39	B(4.75,2.25)	C fibers
Thermal conductivity *k*	0.05–0.14 W/mk	Pig, Human, Monkey, rat	22,30,38,53	U(0.05,0.14)	Dermal & Epidermal
Density *ρ*	1116–1200 Kg/m^3^	Pig, Human, Monkey, rat	22,30,38,53	U(1116,1200)	Dermal & Epidermal
Specific heat *c*	700–950 J/Kg K	Pig, Human, Monkey, rat	22,30,38,53	U(700,950)	Dermal & Epidermal

The reported values of T_threshold_ were estimated at either first spike time or first time if reporting pain or any sensations. The priori density functions are: Normal distribution N, Beta distribution B, and Uniform distribution U.
